# Fragile foundations: succession patterns of bacterial communities in fine woody debris and soil under long-term microclimate influence

**DOI:** 10.1186/s40793-025-00756-9

**Published:** 2025-08-06

**Authors:** Vojtěch Tláskal, Priscila Thiago Dobbler, Jason Bosch, Jörg Müller, Roland Brandl, Claus Bässler, Petr Baldrian, Vendula Brabcová

**Affiliations:** 1 Laboratory of Environmental Microbiology, Institute of Microbiology of the Czech Academy of Sciences, Vídeňská 1083, 14200 Praha 4, Czech Republic; 2 Institute of Soil Biology and Biogeochemistry, Biology Centre of the Czech Academy of Sciences, Na Sádkách 7, 37005 České Budějovice, Czech Republic; 3 Chair of Conservation Biology and Forest Ecology, Biocenter, Julius-Maximilians-Universität Würzburg, 96181 Rauhenebrach, Germany; 4 Bavarian Forest National Park, Freyunger Straße 2, 94481 Grafenau, Germany; 5 Animal Ecology, Faculty of Biology, Philipps-Universität Marburg, 35043 Marburg, Germany; 6 Chair Ecology of Fungi, Bayreuth Center of Ecology and Environmental Research (BayCEER), University of Bayreuth, Universitätsstraße 30, 95440 Bayreuth, Germany

**Keywords:** Decomposition, Deadwood, Bacterial community, Succession, Canopy cover, Microclimate, Temperate forest, Ecology, Fine woody debris

## Abstract

**Background:**

Fine woody debris (FWD; deadwood < 10 cm diameter) is a crucial but often overlooked component of forest ecosystems. It provides habitat for microbial communities and enhances soil fertility through nutrient cycling. This role is especially important in managed forests, which typically have limited deadwood stocks. Climate change is increasing forest disturbances and expanding early successional forests with low canopy cover, yet the effects on microbial communities and related processes remain poorly understood.

**Results:**

In a ten-year canopy manipulation experiment, we examined the decomposition of FWD of *Fagus sylvatica* and *Abies alba*. Increased canopy openness significantly decreased bacterial diversity in decomposing FWD and altered the community composition in surrounding soil. Decomposition time was the main factor shaping bacterial community structure in FWD, with tree species and canopy cover also contributing. We identified bacterial groups involved in carbohydrate degradation, fungal biomass breakdown, and nitrogen fixation. Importantly, bacterial communities in fully decomposed FWD remained distinct from soil communities.

**Conclusions:**

Deadwood decomposition and nutrient cycling are driven by complex ecological interactions. Microbial community dynamics are influenced by the interplay of FWD decomposition stage, tree species, and microclimatic conditions. Bacterial communities, although less frequently studied in this context, appear more stable over time than previously studied fungi. This stability may help sustain decomposition processes and nutrient turnover under the environmental variability associated with global change.

**Supplementary Information:**

The online version contains supplementary material available at 10.1186/s40793-025-00756-9.

## Introduction

Forests are essential for nutrient cycling and serve as a major global carbon sink, storing approximately 45% of the carbon (C) present in terrestrial ecosystems [1, 2]. Soil is the largest terrestrial carbon pool storing roughly 44% of the forest C, while deadwood accounts for another 8%. In natural forests with numerous snags and coarse wood from fallen trees, fine woody debris (FWD, deadwood with a diameter of less than 10 cm) constitutes only a small fraction of the total deadwood stock [3, 4]. In contrast, in managed forests where the major part of trees is typically extracted, FWD represent an important pool of considerable amount. For example, within the management zone of the Bavarian Forest National Park, we measured the amount of fine woody debris (FWD) to be approximately 17.5 m^3^ per hectare [5]. This amount is comparable to the recently reported volume of coarse woody debris found in European forests under various management regimes [6]. These forests are typically subjected to intensive deadwood extraction practices [7]. Therefore, the FWD we observed likely represents the majority of deadwood remaining in such managed forests, reflecting the impacts of strong forest management interventions. FWD shows rapid turnover due to fast decomposition [5, 7]. The carbon flux through FWD is approximately five times faster than that through large deadwood (coarse woody debris, CWD). In total, this flux is equivalent to the carbon flow associated with a volume of roughly 90 m^3^ per hectare of CWD [5]. FWD thus represent a key part of the carbon and nitrogen flow in managed forests.

Deadwood serves as a key source of forest soil nutrient pool [8–11] and plays a crucial role in maintaining forest biodiversity [12, 13], providing both habitat and nutrients for a diverse array of organisms, including microorganisms, such as fungi and bacteria [14–17]. Different deadwood size, turnover dynamic and frequent fragmentation of the FWD creates a variety of habitats and an increased number of habitat patches, leading to a high diversity in detected fungal communities [18] and increase in fungal species richness as a consequence of their competitive life style [19]. Deadwood decomposition is steered by fungi, thanks to broad enzymatic portfolio enabling them to efficiently colonize large sections of wood and decompose it as nutrient source, despite the recalcitrance, fluctuating moisture content and low nitrogen content of this substrate. Studies from forest topsoil and deadwood, however, pointed to the prevalence of bacterial taxa with potential to contribute to decomposition of wood structural compounds like cellulose and hemicellulose [20–22]. These include Bacteroidota (e.g. *Mucilaginibacter*) and Acidobacteriota that incorporate C from ^13^C-labelled cellulose [23, 24]. Similar taxa were characterized as versatile decomposers based on their rich gene toolkit for carbohydrate decomposition [21, 25]. Gammaproteobacteria (e.g. *Hermiinimonas*, *Variovorax*) were also associated with degradation of complex polymers [24, 26, 27]. Contrary to the typically high abundance of Alphaproteobacteria, these decomposers might be actively growing while utilizing labile carbon sources, as they exhibit limited potential for complex carbohydrate decomposition [25]. Acidobacteriota are furthermore reported to be associated with fungal biomass [28], which represents a more accessible substrate from a decomposer's perspective owing to its lower C:N ratio. Indeed, several acidobacterial strains were shown to be able to perform chitin degradation to cover nitrogen and carbon demand [29, 30]. While mycophagy represents one approach how obtain nitrogen at a lower resource cost for microbes, there are other approaches to overcome the recalcitrance of deadwood. Nitrogen might be translocated from surrounding soil [31] or its pool might be enriched in situ by specialized diazotrophic bacteria dwelling in deadwood and performing nitrogen fixation [22, 29]. Increased nitrogen level enables further microbial colonization while it also enables nitrogen immobilization in microbial biomass with potential importance for subsequent soil formation.

Although the majority of studies have targeted the decomposition of coarse wood [17, 32–34] and its influence on surrounding soil, FWD has been shown to be reservoir of fungal forest diversity [5, 18, 19, 35, 36]. The high surface-to-volume ratio of FWD means higher sensitivity of FWD to changes in temperature and moisture [35]. These microclimatic fluctuations are strongly related to both forest management practice and forest stand damage. Importantly, disturbance rates increase with the ongoing global change and damage to canopy cover is expected to be an important factor affecting ecosystem processes [2]. The canopy openness of forests is strongly linked to their temperature buffering capacity [37, 38]. Increased respiration rate [39], elevated summer temperatures, intensive solar radiation, and fluctuating water content are characteristic for forest gaps [40]. The microbial community in FWD is thus subjected to greater fluctuations of these environmental variables. In previous research, focused on characterization of microbial community throughout the lifetime of FWD, the fungal community was shown to be influenced by both tree species of FWD and microclimatic conditions (canopy openness) [5]. In contrast, the bacterial community is typically considered less tightly bound to the deadwood of the preferential tree species [17], although other studies indicate the close relationship [41, 42]. Moreover, bacteria are very sensitive to pH and moisture fluctuations [11, 17, 43].

In this study, we follow up on an experiment where the decomposition successional patterns of the FWD of beech (*Fagus sylvatica*) and fir (*Abies alba*), the two main tree species of the temperate forests of central Europe [44], were followed throughout its decomposition lifetime under open and closed canopies. The aim was to determine the factors affecting bacterial succession during decomposition with the open canopy treatment serving as a proxy of a disturbed ecosystem. Moreover, canopy openness and deadwood source selection allowed us to assess the size and relative importance of the effects of microclimate and the host tree. The frequency of canopy disturbance events has increased in the temperate zone, and they have more pronounced effects in managed forests than in unmanaged forests [45]. Climatic factors are important in influencing fungal community composition [46], while sun exposure and canopy openness influence inner temperature of larger deadwood objects [36], composition of saproxylic beetles [47] and assembly patterns of fungi in FWD [5]. As the wood decomposer strategies may exert significant selection effects [48] determining the bacterial community structure [49], all these environmental factors likely contribute to shape bacterial community development. Deadwood decomposition creates niche differentiation through varying nutrient availability, substrate structure and microbial competition levels [50]. Initial microbial succession in early senescent deadwood favors fungal endophytic and early-arriving groups [50, 51]. This priority effect diminishes in advanced decomposition phase as endophytes and early colonizers struggle to compete under depleted easily available nutrients. As decomposition proceeds, acquiring carbon and nitrogen requires greater microbial investment in enzymes for degrading recalcitrant cellulose and hemicelluloses. Additionally, competition necessitates building protective mechanisms and compensating for extracellular enzyme losses to opportunistic microbes [52]. However, increasing nutrient scarcity (mainly nitrogen) is offset by gradual colonization of nitrogen-rich fungal biomass, diazotrophs activity and nitrogen transport [53]. Together, ongoing deadwood decomposition and local microclimatic conditions create multiple spatial and temporal niches that make microbial succession directional and predictable to some extent.

We hypothesize that the bacterial community will undergo directional succession throughout the decomposition of FWD. This process is expected to involve a decline in endophytic taxa at early decomposition stages, a consistent prevalence of bacterial generalists, and a gradual increase in specialists capable of degrading recalcitrant substrates during the later decomposition stages. Based on previous research on CWD [17], we hypothesize that the bacterial community at the late stage of decomposition with almost decomposed FWD, converges toward soil bacterial community composition. To enable such comparison, we performed characterization of the soil bacterial community at the end of experiment to be able to characterize the overlap of the FWD and soil bacterial communities. In addition to carbon utilization, we expect to record bacterial taxa involved in nitrogen cycling including taxa known for nitrogen fixation and mycophagous bacteria which target fungal biomass for its less restrictive C:N ratio. Previous studies indicate that forest management does not significantly affect soil and rhizosphere bacterial communities in short term scheme [54]. Here, we specifically focused on long-term changes in soil properties and soil bacterial community composition in Bavarian Forest National park, where natural vegetation recovery rate is very slow and we expect detectable changes in soil properties due to the limited primary productivity of open canopy sites.

## Materials and methods

### Study area and experimental design

The study was conducted within the managed zone of the Bavarian Forest National Park, Germany (48.9° N, 13.3° E), which spans approximately 6,000 ha and surrounds the park’s 18,000 ha core zone. This montane region is dominated by mixed forests composed primarily of European beech (*Fagus sylvatica* L.), silver fir (*Abies alba* Mill.), and Norway spruce (*Picea abies* (L.) H. Karst) [35]. The field experiment formed part of a larger, long-term ecological study previously described in detail [5, 55]. In autumn 2011, freshly cut branches of fir and beech were deposited on 64 plots and arranged in a random block design within four spatially independent blocks. The branches (fine woody debris, FWD) had diameters of 3.2 ± 1.3 cm and lengths of 2.7 ± 0.9 m. These branches were taken from trees of the same age that were harvested from the same forest stand. The branches were further randomly selected from the prepared material and distributed across study sites to mitigate the potential effect of communities of fungal endophytes inhabiting individual branches on microbial community development. Each block contained randomly located sets of plots with either fir or beech branches or both. Deadwood mixtures of fir and beech were used to simulate tree species diversity within forest stands. Within each block, two replicate plots for each treatment (fir, beech, or mixed) were established under both open and closed canopy conditions (Supplementary Fig. 1). Canopy openness served as a proxy for varying stand microclimatic conditions. [13, 38, 55, 56]. Open canopy conditions were created by clearing approximately 0.1 ha of forest, removing both live and dead trees. To prevent excessive shading from the surrounding herbaceous vegetation, particularly grasses, these plots were mowed once annually during the growing season, following previously established protocols [57, 58]. The daily peak temperatures of the deadwood surfaces in summer were measured in the open and closed plots. The mean values were much higher in open canopy plots (~ 30 °C) than in the closed canopy plots (~ 15 °C) [59]. All experimental plots were sampled annually each September or October from 2012 to 2018, and once more in 2021 following a three-year decomposition period, bringing the total duration of the decomposition experiment to ten years. Soil samples were collected in 2022.

### Sampling, sample processing and analysis

One composite FWD sample was collected from each selected branch by performing two vertical drillings at the center of the branch using an electric drill fitted with a 10 mm diameter auger. Drillings were made across the full diameter of the branch, with sampling points evenly spaced along its length, while avoiding areas near the branch ends. The auger was sterilized between each drilling using ethanol and flame to prevent cross-contamination, although potential DNA carryover cannot be fully excluded given ethanol’s limited efficacy. The dust from all drilling points was collected in sterile plastic bags and frozen within a few hours after drilling. In total, 2 beech or 2 fir samples were taken from each block from plots containing only beech or fir, and 2 beech and 2 fir samples were taken from plots containing mixed deadwood, resulting in 4 beech and 4 fir composite samples per canopy type. In total, 64 samples were collected each year (Supplementary Fig. 1), yielding 512 samples available for the present study.

Drilled samples were weighed and freeze-dried in the laboratory to assess deadwood dry mass. Next, it was milled using an Ultra Centrifugal Mill ZM 200 (Retsch, Germany), and the resulting fine powder was used for the subsequent analyses. Deadwood pH was measured after mixing with distilled water (1:10 w: vol). The wood carbon and nitrogen contents were measured using an elemental analyzer in an external laboratory of the Institute of Botany of the Czech Academy of Sciences, Průhonice, Czech Republic, as described previously [60]. Carbon was measured using sulfochromic oxidation, and the nitrogen content was estimated by sulfuric acid mineralization with the addition of selenium and sodium sulfate and conversion to ammonium ions, which were measured by a segmented flow analyzer (SFA), Skalar. Fungal biomass was quantified by extracting total ergosterol using 10% KOH in methanol, followed by analysis via HPLC [61].

Soil from all sampling sites was collected during a single campaign in September 2022. Sampling points were located in close proximity (approximately 1–1.5 m) to the tested FWD objects. At each location, plastic soil cores (4 cm in diameter) were hammered into the ground at the central point and at positions 0.5 m to the north, south, west, and east. Entire soil cores were placed into plastic bags and immediately transported to the laboratory, where they were stored at 4 °C for no longer than 24 h before processing. During processing, the litter layer was removed and discarded. The top 10 cm of mineral soil from each core was combined, homogenized, and sieved through a 5 mm mesh to create a composite sample. These homogenized soil samples were immediately frozen, freeze-dried, and stored at − 20 °C until further analysis. All subsequent analyses were conducted using the same protocols as those applied to the deadwood samples.

### Extraction and analysis of environmental DNA

Total genomic DNA was extracted from 200 mg of freeze-dried material using the NucleoSpin Soil Kit (Macherey–Nagel, Germany), following the manufacturer’s protocol. In brief, cells were lysed using SL1 lysis buffer with the addition of Enhancer SX. Samples were homogenized using a FastPrep-24 instrument (MP Biomedicals, Santa Ana, USA) at 5 m s⁻^1^ for 2 × 30 s. In the final step, DNA was eluted from the spin columns with 50 µl of deionized water. One extraction was performed per sample. [62].

For the bacterial community analysis, PCR amplification of the prokaryotic hypervariable V4 region of the 16S rRNA gene was performed using barcoded 515 F and 806R primers [63] in triplicate PCRs per sample as described previously [17]. PCRs contained 5 µl of 5 × Q5 reaction buffer, 1.5 µl of BSA (10 mg ml^−1^), 1 µl of each primer (0.01 mM), 0.5 µl of PCR Nucleotide Mix (10 mM each), 0.25 µl of Q5 High Fidelity DNA polymerase (2 U µl ^−1^, New England Biolabs, Inc.), 5 µl of 5 × Q5 HighGC Enhancer and 1 µl of template DNA (approx. 50 ng µl^−1^). Cycling conditions were 98 °C for 30 s, 25 cycles of 94 °C for 10 s, 56 °C for 30 s, and 72 °C for 20 s, and a final extension at 72 °C for 2 min. Negative and positive amplification controls were used to avoid contamination errors and to prove the efficiency of the PCR, but these controls were not sequenced within this project. PCR triplicate reaction products were pooled and purified (MinElute PCR Purification Kit, Quiagen), and amplicon libraries prepared with the TruSeq DNA PCR-Free Kit LP (Illumina) were sequenced in house on the Illumina MiSeq (2 × 250-base reads).

The amplicon sequencing data were processed using the pipeline SEED 2.1.3 [64]. Briefly, paired-end reads were merged using fastq-join [65]. Chimeric sequences were detected using Usearch 11.0.667 [66] and deleted, and sequences were clustered using UPARSE implemented within Usearch 8.1.1861 [67] at a 97% similarity level. The most abundant sequence was selected from each cluster, and the closest hits at the species level were identified using BLASTn against an edited version of the SILVA 138.1 database including mitochondria and chloroplasts. Where the best hit showed lower similarity than 97% with 95% coverage, the best genus-level hit was identified. The sequences used in all community structure analyses were rarefied at 11,400 sequence (median of sequence count for all samples) to reduce bias from uneven sequencing depth while retaining as much data as possible. Sequences from both FWD and soil samples were processed together, with the dataset subsequently split as needed for specific analyses. For species-level analyses, OTUs assigned to the same species were merged, while remaining OTUs were grouped at the genus level based on the best BLAST hit and labeled as “sp.” Only sequences identified as bacterial were included in the final analysis. Sequencing data have been deposited in the NCBI SRA database under BioProject accession number PRJNA1228314 and in Zenodo repository (10.5281/zenodo.14900894).

### Data processing and statistics

*Successional timing* (expressed in years) or so called *temporal niche position* was defined as the average position of a taxon in succession considering its relative abundance over time according to the previously published formula [68], but the time in year is used for calculation. *The duration of occurrence* was defined as the time span covering 90% of the taxon relative abundance as defined and calculated previously [68]. Tree or canopy specificity was defined as the strength of association of the taxon with one particular tree or canopy type and calculated as the sum of abundances in beech deadwood (closed canopy deadwood) divided by the sum of abundances in all samples. A specificity value of 1 indicates a taxon found exclusively on beech deadwood, while a value of 0.5 denotes equal abundance on both beech and fir deadwood. A value of 0 signifies a taxon exclusively associated with fir deadwood. Regarding canopy openness, a value of 1 corresponds to taxa found only under the closed canopy, whereas a value of 0 indicates taxa exclusively found under the open canopy. Taxa with specificity values between 0.95 and 1.0 were classified as beech-specific (or closed canopy-specific), while those between 0.00 and 0.05 were classified as fir-specific (or open canopy-specific).

Statistical analyses were performed in R [69]. To assess differences in bacterial community composition, two-dimensional non-metric multidimensional scaling (NMDS) ordination was performed on Bray–Curtis dissimilarities calculated from Hellinger-transformed relative abundance data, using the metaMDS function from the *vegan* package [69, 70]. Variables were fitted to the ordination diagram as vectors with 999 permutations and included pH, as well as the carbon and nitrogen and ergosterol contents. Diversity estimates (Shannon–Wiener index, OTU richness, Chao 1 and evenness) were calculated for a dataset containing the relative abundance of 2100 randomly selected sequences from each sample in SEED 2.1.3. [64]. Differences in the environmental variables (pH, carbon, nitrogen, C:N, ergosterol and water contents) were assessed using a linear mixed model with log-transformed data (LMM, function lmer). For the LMM, the effect of explanatory variables, i.e., tree species, canopy openness and their interaction, over decomposition time were tested by considering time and plot identifiers (the same plots were repeatedly measured over time, the objects are nested in block/plot) as random effects. One-way and two-way PERMANOVA tests with 9999 permutations were conducted to evaluate the effects of treatments on bacterial community composition. Spearman rank correlations were used to assess relationships between variables. Variation partitioning analyses of Hellinger-transformed OTU abundances were performed to quantify the variance explained by tree species and canopy openness for the entire dataset. Additionally, for each tree species separately, the effects of decomposition length, canopy openness, and wood chemistry parameters (nitrogen, carbon, C:N ratio, and pH) were evaluated (using the varpart function from the *vegan* package [70]). The significance of variance components was assessed with Monte Carlo permutation tests. Differences with *P* < 0.05 were considered statistically significant.

## Results

### Properties of fine woody debris during ten years of decomposition

**Fig. 1:**
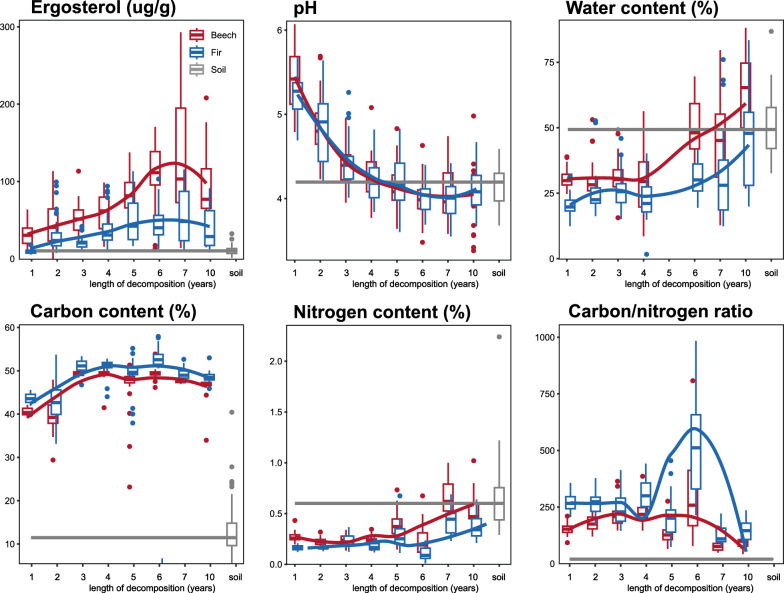
Fungal biomass and changes in physicochemical compositions of beech and fir fine woody debris during decomposition and in surrounding soil. The differences in canopy openness is not considered. Data represent the mean ± SE of 8 samples per treatment and timepoint (years of decomposition) for FWD in red (beech) and blue (fir) and 46 samples per treatment for soil (grey)

The physicochemical properties of decomposing fine woody debris (FWD) over a decade revealed dynamic development similar to the early decomposition phases [5]. Carbon content increased during the initial stages of decomposition but seems to stabilize thereafter (LMM: χ^2^ = 2.37, *P* = 0.12). Fir (*Abies alba*) FWD had significantly higher carbon content than beech (*Fagus sylvatica*) (LMM: χ^2^ = 49.85, *P* < 0.001, Fig. 1), while canopy cover showed no significant effect (data not shown). Nitrogen content increased gradually over time (LMM: χ^2^ = 5.26, *P* = 0.02), with beech FWD containing more nitrogen than fir (LMM: χ^2^ = 52.39, *P* < 0.001, Fig. 1). Nitrogen was generally lower under canopy gaps, with a significant canopy effect only for fir deadwood (LMM: χ^2^ = 4.35, *P* = 0.04). From year 7 onwards, nitrogen content approached soil levels. The C:N ratio declined over time, though this trend lacked statistical significance due to high variability (Fig. 1). FWD pH decreased during the first seven years, reaching a minimum, followed by a slight increase by year 10 (LMM: χ^2^ = 8.49, *P* = 0.00, Fig. 1). pH was higher under open canopy (LMM: χ^2^ = 6.68, *P* = 0.01) but did not differ between tree species. Water content rose during later decomposition stages (LMM: χ^2^ = 11.88, *P* < 0.001), with beech FWD consistently more moist than fir (LMM: χ^2^ = 47.32, *P* < 0.001, Fig. 1). Fungal biomass (ergosterol content) increased during the first 6–7 years before declining (LMM: χ^2^ = 7.50, *P* = 0.00), with beech and closed canopy conditions supporting approximately double ergosterol levels compared to fir and open canopy (LMM: χ^2^ = 76.78 and 94.59, *P* < 0.001, Fig. 1). These trends suggest that the depletion of accessible nutrients characterizes advanced decomposition stages.

### Long-term changes in soil physicochemical properties caused by canopy openness

**Fig. 2:**
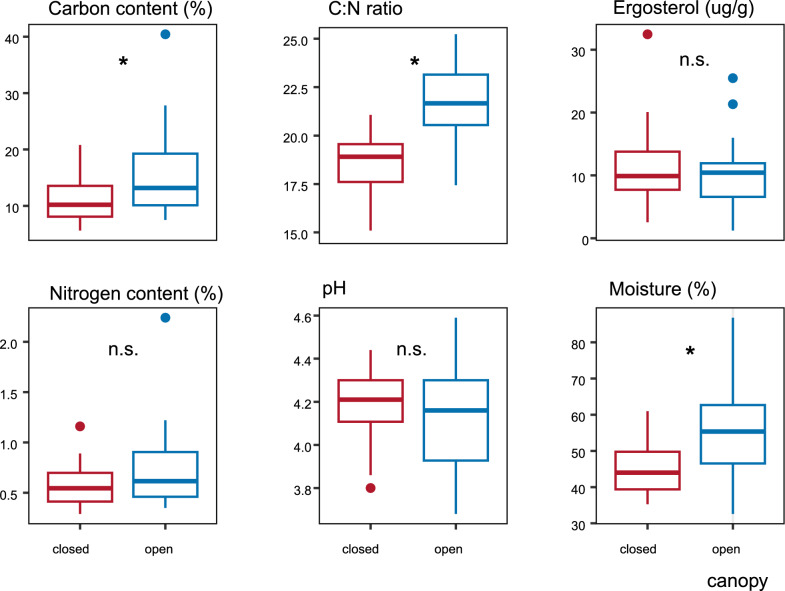
Fungal biomass and changes in physicochemical compositions in soil caused by 10 years of development under different microclimatic condition caused by forest stand canopy openness. Boxplots show data based on 23 soil samples per treatment

Soil properties showed significant long-term changes related to canopy openness (Fig. 2). The soil C:N ratio increased from 18.7 under closed canopy to 21.2 under open canopy (ANOVA: F = 35.36, df = 1, *P* < 0.001), despite spatial variability (ANOVA: F = 1.48, df = 3, *P* = 0.24). Soil carbon content increased significantly following canopy opening (ANOVA: F = 9.05, df = 1, *P* < 0.001) and was only marginally affected by site. Soil nitrogen content and fungal biomass varied primarily by site and were less influenced by canopy openness. Soil moisture increased significantly under open canopy (ANOVA: F = 3.03, df = 1, *P* = 0.04) and was affected by both canopy and site factors (Supplementary Table 1). Soil pH remained constant regardless of canopy or site. All soil parameters differed significantly from values measured in decomposing FWD (ANOVA: *P* < 0.002, Fig. 2 and Supplementary Table 1), highlighting distinct physicochemical profiles.

### Impact of canopy openness on bacterial community composition and diversity in soil

**Fig. 3:**
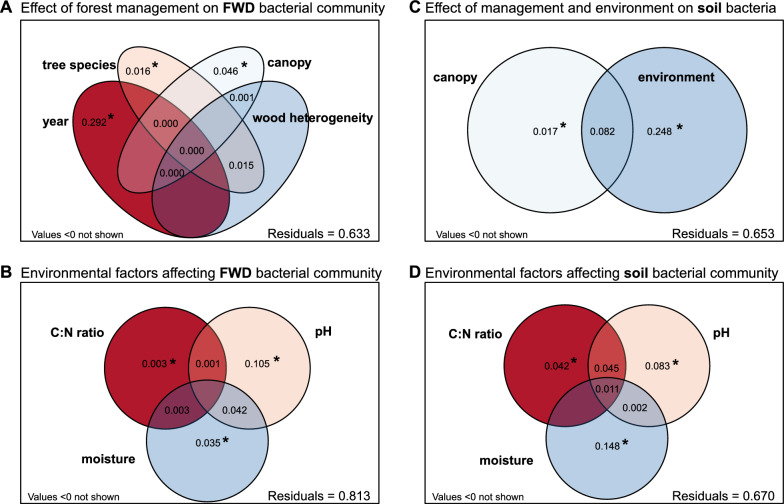
Venn diagram representing the results of variation partitioning analyses on Hellinger-transformed OTU abundances. **A** Treatment specific effect: length of decomposition, tree species, canopy cover and deadwood heterogeneity of origin on site calculated using all FWD samples, **B** Effect of environmental factors on FWD bacterial community composition calculated using all FWD samples, **C** Effect of canopy openness and environmental factors on soil bacteria calculated using all soil samples, **D** Effect of selected environmental factors on soil bacterial community composition calculated using all soil samples

Bacterial diversity (species richness and Chao-1 index) was significantly higher in soil than in FWD (Kruskal–Wallis test, *P* = 0.000). Soil diversity was not significantly affected by canopy openness (ANOVA: species richness F = 1.53, P = 0.22; Chao-1 F = 1.06, *P* = 0.319). However, soil bacterial community composition was significantly influenced by canopy cover and environmental factors (PERMANOVA: F = 6.97 and 2.22, *P* = 0.00 for both; Fig. 3). Canopy cover explained 1.7% and environmental factors 24.8% of community variability (variation partitioning, *P* = 0.002 and 0.00). Among environmental factors, soil moisture explained the largest variability (14.8%, *P* = 0.001), followed by soil pH (8.3%, *P* = 0.00) and C:N ratio (4.2%, *P* = 0.00).

**Fig. 4:**
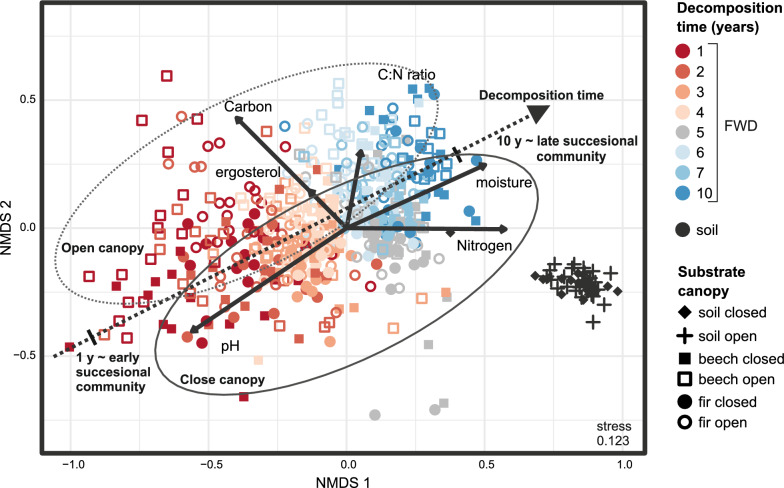
Non-metric multidimensional scaling (NMDS) of the bacterial community in decomposing fine deadwood of beech and fir, as well as surrounding soil, under different microclimatic conditions in a natural temperate forest. The analysis is based on Euclidean distances of Hellinger-transformed relative abundances. Vectors represent the potential influence of environmental variables. The dashed line indicates the direction of temporal development. Two ellipses highlight the distribution of samples from open canopy (dotted line) and closed canopy (solid line) conditions. Only OTUs with relative abundances exceeding 0.5% in at least three samples were included

**Fig. 5:**
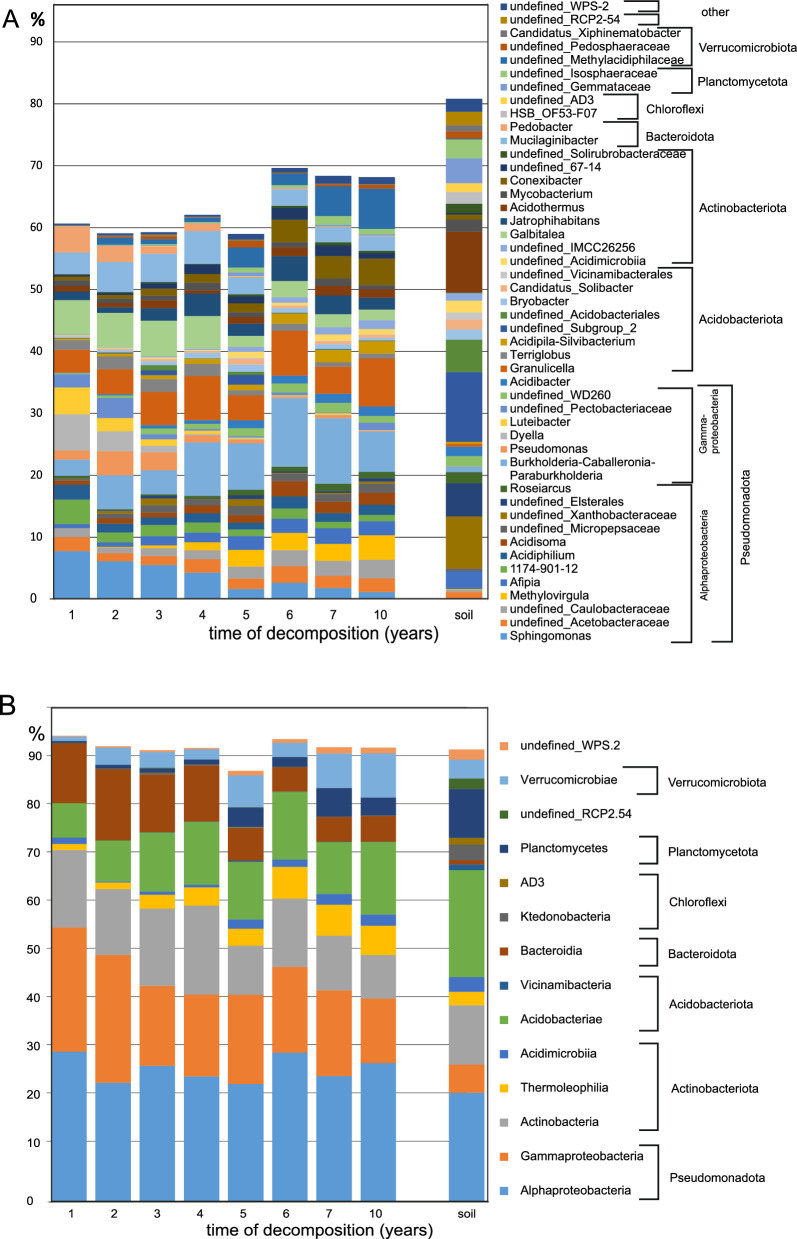
Abundant bacterial genera (**A**) and phyla (**B**) in decomposing fine beech and fir deadwoods and soil in a natural temperate forest. The data represent the means of 32 samples of FWD or 46 samples of soil. Bacterial genera and phyla with average abundances over 1% are included

Soil and FWD bacterial communities differed significantly (PERMANOVA: F = 151.3, df = 1, *P* = 0.001). Canopy-related differences in soil were also significant but appeared less distinct in the NMDS plot due to strong successional turnover (Fig. 4). Dominant soil bacterial phyla included Pseudomonadota (28.5%, mainly Alphaproteobacteria, 22.7%), Acidobacteriota (22.2%), and Planctomycetota (10.1%) (Fig. 5). Dominant soil genera (e.g., *Acidobacterium*, *Acidothermus*, *undefined Xanthobacteraceae bacterium*, *undefined Elsterales bacterium*, and *undefined Acidobacteriales bacterium*) each exceeded 5% relative abundance and together accounted for 85% of total soil bacterial abundance, but only 30% in FWD (Supplementary Fig. 2). Most abundant taxa (> 0.5%) were not canopy-specific, but *Burkholderia-Caballeronia-Paraburkholderia* group was more abundant under closed canopies, while *Occallatibacter* and *Bacillus* were prevalent under open canopies (> 75% of sequences; Supplementary Table 2).

### Bacterial community compositions in the different types of fine woody debris

Bacterial diversity in FWD was significantly lower than in soil but higher under closed canopy and in fir debris (Kruskal–Wallis test, *P* = 0.00; Fig. 4). In fir FWD, species richness peaked under open canopy in years 3 and 5 before declining, though it remained above year 1 levels (Supplementary Fig. 3). Diversity varied widely across FWD samples during decomposition.

Bacterial communities in beech and fir FWD underwent continuous change over time (PERMANOVA: F = 166.5, *P* = 0.001; Fig. 4), though less markedly than fungal communities [5]. Canopy cover and deadwood origin also significantly influenced bacterial composition (F = 34.43 and F = 22.93, *P* = 0.001), though these effects were not clearly visible in the NMDS plot (Fig. 4). In contrast, deadwood heterogeneity had no significant impact (F = 0.87, *P* = 0.84).

Temporal succession emerged as the dominant driver, explaining 29.3% of the variability in bacterial community composition (PERMANOVA: F = 32.63, *P* = 0.00; Fig. 3). Canopy cover and tree species accounted for smaller but significant portions (4.7% and 3.1%, respectively; Fig. 3). Despite the expectation that soil bacteria respond to physicochemical conditions, environmental factors alone explained only 1.3% of the variation and were not statistically significant (F = 1.28, *P* = 0.126). When combined with time, they accounted for 17.4%, with time alone explaining 11.7%. Still, 62% of the variation remained unexplained, highlighting the complexity of bacterial community dynamics in decomposing FWD.

**Fig. 6:**
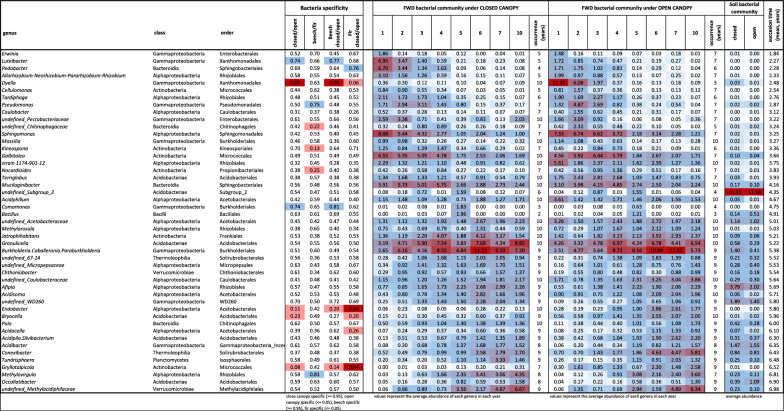
Successional development of the bacterial communities in beech *(Fagus sylvatica)* and fir *(Abies alba)* fine deadwoods decomposing under closed and open canopies in a natural temperate forest. All taxa with abundances above 1% in 3 yearly observations were listed

FWD bacterial communities were dominated by Pseudomonadota (46.7%), mainly Alphaproteobacteria (27.5%) and Gammaproteobacteria (19.2%), followed by Actinomycetota (13.6%) and Acidobacteriota (11.7%). These dominant phyla showed clear successional trends toward soil-like composition: Pseudomonadota and Bacteroidota declined, Acidobacteriota increased, and Actinomycetota fluctuated. Verrucomicrobiota and Planctomycetota became more prominent over time, though FWD communities remained distinct from soil even after 10 years (Fig. 6, Supplementary Fig. 4).

At the genus level, dominant taxa grouped into early (years 1–3) and late (years 4–10) successional phases. Early-phase dominants included *Sphingomonas* (6.4%), *Galbitalea* (5.6%), *Granulicella* (4.4%), *Mucilaginibacter* (4.3%), *Burkholderia*-*Caballeronia*-*Paraburkholderia* (4.0%), *Dyella* (3.4%), *Pseudomonas* (2.8%), and *Pedobacter* (2.7%). In later stages, the community shifted toward *Burkholderia*-*Caballeronia*-*Paraburkholderia* increased to 8.8%, alongside *Granulicella* (6.1%), and other genera such as *Methylacidiphilaceae* (3.4%), *Mucilaginibacter* (3.1%), *Conexibacter* (2.9%), and *Jatrophihabitans* (2.9%). Most taxa persisted over time, with faster turnover limited to some early-stage groups (Fig. 6). Dominant taxa were generally not specific to tree species or canopy cover, with a few exceptions showing preference (Supplementary Table 2). Succession times did not differ significantly between beech and fir FWD or between canopy conditions (Fig. 6).

## Discussion

Our study highlights key factors influencing fine woody debris (FWD) decomposition and associated bacterial communities, including the effects of microclimatic conditions caused by canopy openness on surrounding soil bacterial communities. We observed a rapid initial decomposition phase characterized by the increase in bacterial diversity and presence of early-occurring bacterial taxa followed by a slowdown, linked to pH stabilization. Canopy openness affected FWD and soil properties but had limited long-term effects on fungal biomass present in FWD, while bacterial diversity in FWD and soil responded primarily to substrate characteristics rather than canopy conditions. Bacterial community succession in decomposing FWD revealed a continuous shift, with a few canopy- and tree-specific taxa. Nonetheless, even in the highly advanced decomposition stage, bacteria inhabiting decomposing FWD—spanning a broad range of functional roles—formed a distinct community compared to the surrounding soil. These findings underscore the complex interplay between canopy structure, soil properties, and microbial community dynamics in forest ecosystems*.*

### Fine woody debris decomposition

The extension of the FWD decomposing experiment [5] allowed observation of complete FWD lifespan. While decomposition progressed rapidly in early years, the rate slowed after seven years, indicated by a gradual decrease in ergosterol content and an increase in pH values. This pattern may reflect decline in fungal wood decomposer activity typical for coarse deadwood [62, 71], as fungi actively lower pH through the production of organic acids. Higher ergosterol content and moisture levels in beech FWD are consistent with faster decomposition of beech deadwood, as previously reported [71–73]. The increase in nitrogen content in the late decomposition phase, partially due to the accumulation of nitrogen-rich microbial biomass and biological nitrogen fixation, suggests a gradual convergence with soil nutrient profiles [9].

Despite variations in decomposition time, changes in deadwood chemistry were largely consistent with initial wood composition, leading to relatively uniform decomposition process across different deadwood origins and microclimatic conditions. Fluctuations in FWD pH, moisture content [75] and fungal community shifts [48, 49], were likely key factors influencing bacterial assembly, as these parameters strongly predict bacterial composition on decomposing deadwood.

### Long-term changes in soil physicochemical properties caused by canopy openness

Canopy openings influence forest microclimates [37, 38] and affect soil properties, especially carbon content and nutrient availability [76]. These effects vary with gap size, age, and forest stand characteristics [77]. Contrary to expectations, we observed an increase in total soil carbon and C:N ratio, likely due to altered organic matter inputs and the slow recovery of forest floor vegetation. Dense grass layer and absence of clear structured organic layer were frequent under open canopy, contrasting with closed forest conditions. Increased soil moisture was also observed in other forest clearcutting experiments (data not published). Despite changes in soil conditions, fungal biomass remained unaffected by canopy opening in the long term. This aligns with previous findings showing that fungal biomass declines immediately after clear-cutting, but recovers rapidly within 2 years in regenerating forests [78, Awokunle-Holla, in preparation]. Microbial activity, as indicated by soil CO₂ efflux, has been shown to be influenced more by soil properties than microclimatic factors [79], though increased respiration has been reported in some gap studies [39].

Higher soil moisture in gaps, detected in our study and repeatedly reported previously [40], likely results from reduced transpiration-driven water loss. This has important ecological implications, as climate change induced droughts severely impact forests [80]. However, small canopy gaps with reduced tree water uptake may serve as refugia for moisture-sensitive soil organisms. While fungi are generally more drought-resistant, bacterial communities are more vulnerable to prolonged moisture deficits [81]. Soil pH plays a pivotal role in shaping microbial communities and influencing nutrient cycling and organic matter decomposition, but no significant changes in soil pH were detected in our study or in a recent meta-analysis on canopy gaps [76]. These observations underscore the complex interactions between canopy structure, soil conditions, and microbial communities in forest ecosystems.

### Drivers of bacterial community composition in fine deadwood and soil, effect of canopy openness

Bacterial diversity reached its peak between the third and the fifth year of decomposition, showing the steep increase after the initial two years before declining towards the end of the decomposition process. The low diversity values during initial decomposition can be attributed to a less developed microbial community that utilizes labile carbon sources and includes potential plant pathogens known from living trees like genus *Erwinia*, [82] and taxa typically detected in early decomposition phase of CWD (genera *Sphingomonas*, *Sodalis* and *Pseudomonas*, [25]. The priority effect, which favors already present taxa, likely shapes the initial decomposition phase [51, 83]. The middle stage of decomposition is characterized by increasing bacterial diversity [84, 85], suggesting that the complex community might be reaching the maximum carrying capacity of FWD as a growth substrate. During the final decomposition stage, the bacterial community shows decreasing diversity and structurally approaches the composition of soil communities. This convergence occurs as FWD disintegrates and mixes with the surrounding soil, leading to increasingly similar habitat variables and, consequently, similar communities.

Despite the convergence trend, structural differences between FWD and soil bacterial communities remained significant throughout the decomposition process in our experiment, with FWD communities consistently showing relatively high diversity, yet significantly lower than soil bacterial communities. This lower diversity can be attributed to less favorable conditions for bacterial growth in deadwood, including high C:N ratios, lower pH and likely increased competition for nutrients with fungi in a habitat lacking carbon input through rhizodeposition [74, 86]. While soil serves as a reservoir of bacterial diversity for deadwood colonization [87], the selective pressures within deadwood drive the community through specific developmental stages. The decomposition process itself continuously influences pedological properties and microbial community in the upper horizons of underlying soil, creating an interplay between these two physically connected habitats [72].

Similarly to fungal diversity [5], fir deadwood supported higher bacterial diversity than beech, extending our understanding of substrate-specific community development during FWD decomposition. While previous studies have documented comparable or lower bacterial richness in beech compared to other tree species [17, 75], the diversity patterns in fir remained unexplored. The contrasting responses of bacterial and fungal communities to canopy conditions—with bacterial diversity flourishing under closed canopy while fungal diversity in fir FWD increased with canopy opening [5]—suggest distinct niche differentiation mechanisms. Bacterial sensitivity to drought [81] likely limits community diversity under open canopy conditions, where higher sun irradiation increases FWD desiccation stress. Despite the observed influence of tree species and canopy openness on community structure, the major bacterial taxa in FWD displayed remarkable habitat generalism, showing no strong preferences for either tree species or canopy conditions. This points to a more complex interplay of factors shaping bacterial communities in FWD beyond tree species and canopy effects. The residual unexplained variability in bacterial community structure might be attributed to unmeasured environmental parameters—such as temperature fluctuations or microfaunal activity—and probably to a larger extent, to processes induced by fungal community composition. Previous research on deadwood decomposition in natural forests showed high confidence in predicting bacterial community structure based on fungal composition [49]. In other words, fungal communities have high potential to modulate bacterial structure. FWD rich in fungal biomass provides a suitable habitat for such relationships.

The pattern of environmental response extended to the soil bacterial communities, where canopy openness induced subtle yet significant structural changes likely through altered forest floor dynamics, including shifts in vegetation, litter input, and root exudate profiles [88]. The absence of canopy-specific associations among dominant soil bacterial taxa, combined with their stronger response to abiotic factors such as soil moisture, C:N ratio, and pH [89], indicates that bacterial community assembly in both FWD and soil is primarily driven by fundamental habitat conditions rather than canopy-related variables, confirming the previous results from forest clearcuts [54].

### Generalists versus specialists

The genus *Granulicella* (Acidobacteriota) and *Mucilaginibacter* (Bacteroidota) showed genomes containing rich toolkit of CAZymes which suggests their broad substrate specificity [25, 90]. Indeed, these taxa were identified at high relative abundances throughout the whole FWD decomposition which might indicate that they may modulate their metabolism to maintain high population density even during the changes in substrate availability. The role of Acidobacteriota in the decomposition was confirmed by the fact that their carbohydrate-active enzymes were widely expressed on coarse deadwood [20]. In addition to Acidobacteriota, potential for versatility in carbohydrate utilization was shown in a global genome comparison also for Planctomycetota and Verrucomicrobiota [21] which appeared as *Tundrisphaera* (Planctomycetota), *Chthoniobacter* and *Methylacidiphilaceae* bacterium (Verrucomicrobiota) in the advanced FWD decomposition phase.

In contrast to generalists, several taxa showed preference for a specific phase of FWD decomposition. This includes *Erwinia* (Gammaproteobacteria) known as a potential pathogen from live plant tissues [82] and from association with insect pests [91]. Their high abundances at the beginning of decomposition might represent a diminishing community, which is not adapted for long-term survival in decomposing wood.

*Luteibacter* displayed the preference for the early FWD decomposition and was shown to degrade cellulose in soil [92]. In a pure culture, *Luteibacter* carbohydrate utilization was performed mainly through the cell-associated enzymes [93] which might prevent resource loss through diffusion of decomposition products but also represents disadvantage in the advanced decomposition when proximal resources might be exhausted. *Pedobacter* abundant at the beginning of FWD decomposition was shown to degrade simple compounds [26] and thus this taxon might be outcompeted in later stages of FWD decomposition.

### Further possible bacterial functions

Despite less pronounced bacterial potential to degrade complex compounds in comparison with fungal activity owing to a limited ability to colonize and change large patches of the substrate, bacteria still hold potential to contribute to the process of decomposition with decisive roles. Their roles are represented by functional traits described in the following paragraphs. While 16S rRNA provides limited functional resolution to identify specific bacterial functions, bacterial taxa identified here have been repeatedly observed in association with decomposing wood; Acidobacteria, Alphaproteobacteria, Gammaproteobacteria, Actinobacteria, and Bacteroidetes were previously identified in deadwood by 16S rRNA sequencing and cultivation [16, 25]. Members of the Alphaproteobacteria cultivated from deadwood showed lower potential for carbohydrate utilization as seen in their limited CAZyme gene content and thus narrow spectrum of carbohydrates which they can utilize [25].

On the other hand, Alphaproteobacteria together with Gammaproteobacteria are able to utilize C1 compounds. Most importantly, they can oxidize methane and subsequently methanol, both of which are produced during deadwood decomposition [94]. Part of the carbon from these compounds is also assimilated and thus stabilized which prevents its loss from the system. The potential taxa feeding on C1 compounds in decomposing FWD are represented by *Methylorosula* (Alphaproteobacteria) [95], *Methylovirgula* (Alphaproteobacteria) [96] and *Methylacidiphilales* (Verrucomicrobiota), although the methanotrophic abilities of the latter probably depend on the habitat [97, 98].

While deadwood is carbon-rich substrate, its nitrogen limitation makes its colonization and decomposition difficult. Community of diazotrophs alleviate nitrogen limitation in deadwood [32] which has the strongest effect at the beginning of decomposition. In situ expression of nitrogen-fixation genes was shown to be strong on coarse deadwood [29]. Transcripts of nitrogen fixation genes mapped to the genomes/metagenome-assembled genomes have unveiled potential diazotrophs which include also genomes of cultivated enterobacteria from the genus *Sodalis* (identified as Pectobacteriaceae bacterium in decomposing FWD) [99]. Here *Sodalis* appears throughout the decomposition of FWD with the highest values at the beginning of FWD decomposition, which is in line with its putative role in nitrogen fixation. In terms of FWD decomposition, the initial years were characterized by low nitrogen content, which increased only after four years as nitrogen enrichment by diazotrophs, and nitrogen retention in microbial, mainly fungal, biomass took place.

Recycling of microbial biomass is among the major factors sustaining bacterial growth in forest soil [26, 27]. Given the high amount of fungal biomass in deadwood, mycophagous bacterial lifestyle can represent a successful strategy in obtaining the energy and covering carbon and nitrogen demand. Several chitinolytic bacteria were identified in the phylum Acidobacteriota [24, 100] and members of this group were identified as either being present throughout the FWD decomposition (genus *Granulicella*, *Terriglobus*) or at the advanced phase of decomposition (*Bryocella*, *Silvibacterium*, *Occallatibacter*).

## Conclusions

This study demonstrates that microclimatic changes induced by forest canopy gaps significantly influence soil bacterial communities and those involved in the decomposition of fine woody debris (FWD). Although FWD bacterial communities originate from the soil, they undergo rapid and dynamic development, shaped in part by microclimatic factors. As FWD plays a crucial role in nutrient flow, particularly in managed forests, disturbances to the canopy could disrupt nutrient dynamics and ecosystem functions. While fungi are the primary decomposers of dead plant biomass, their function is heavily influenced by substrate quality and microclimatic conditions. In contrast to fungi, bacteria appear to represent a more stable component of the microbial community, even in such fluctuating environments. Environmental factors not only impact the structure of microbial communities but can also modulate the metabolic activities of their members [101].

A key limitation of this study lies in the functional inferences based solely on 16S rRNA gene data; while associations with traits such as nitrogen fixation or mycophagy provide valuable ecological insights, they remain speculative without direct evidence of metabolic activity. The absence of functional gene or expression data limits confirmation of the actual roles played by bacterial taxa. Therefore, future research should integrate approaches such as metagenomics, metatranscriptomics, or stable isotope probing to achieve a more robust understanding of microbial function as applied recently [27, 29]. Such studies should particularly focus on functional gene activity related to nutrient cycling, microbial resilience, and bacterial–fungal interactions across varying canopy conditions. Additionally, examining disturbance-specific bacterial functions and their impact on carbon dynamics will be crucial for advancing sustainable forest management.

## Supplementary Information

Additional file 1.

Additional file 2.

Additional file 3.

Additional file 4.

Additional file 5.

Additional file 6.

## Data Availability

Sequencing data have been deposited in the SRA database under BioProject accession number PRJNA1228314 and in Zenodo repository (10.5281/zenodo.14900894).
