# Supplementary material for: Fragile foundations: succession patterns of bacterial communities in fine woody debris and soil under long-term microclimate influence
Source: Environ Microbiome. 2025 Aug 6;20:101. doi: 10.1186/s40793-025-00756-9 (PMC12330196; doi:10.1186/s40793-025-00756-9)
Supplement: Supplementary file 1 — Additional file 1. [file 40793_2025_756_MOESM1_ESM.pdf]

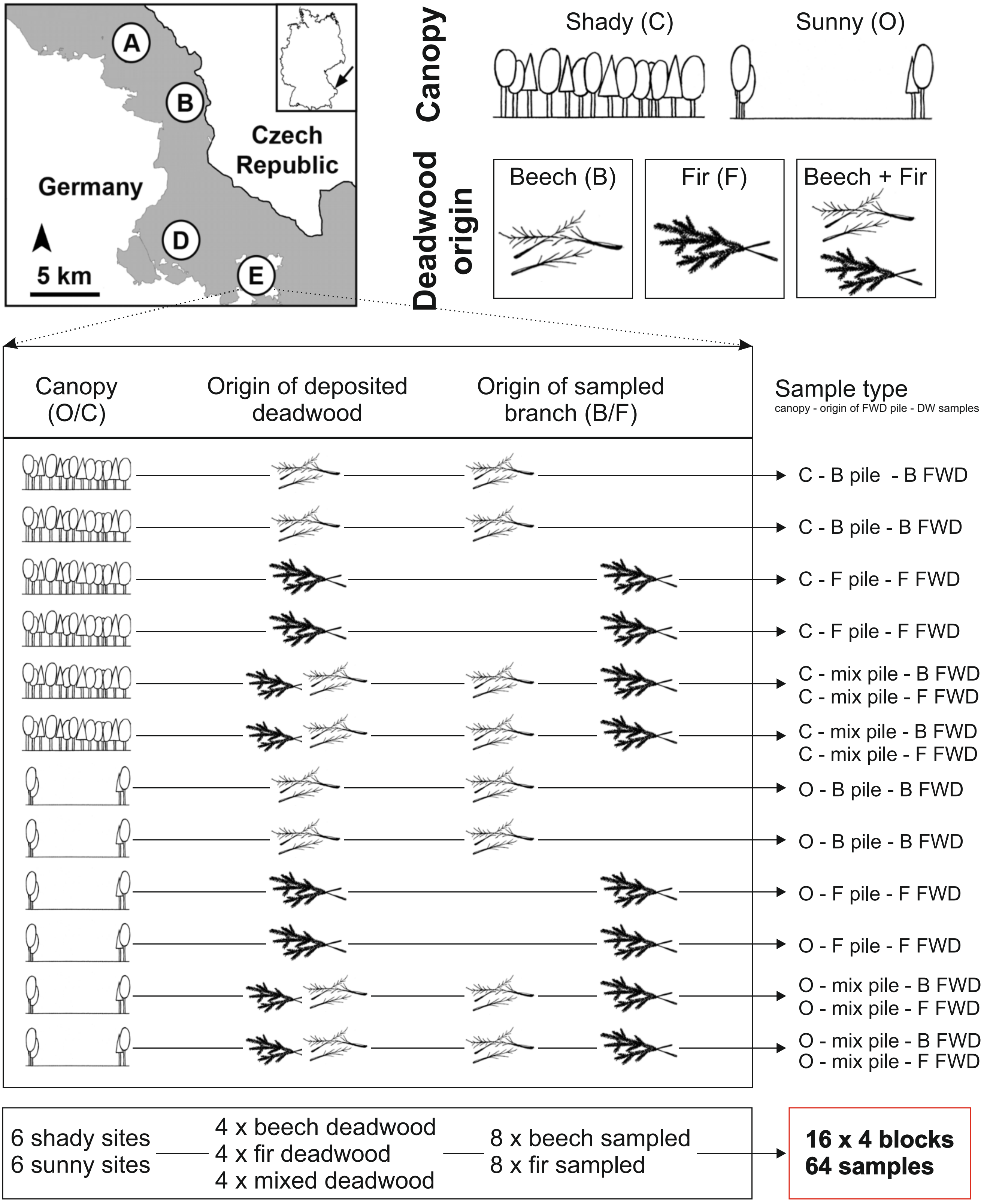

A
B
Czech Republic
Germany
D
E
5 km
Canopy
Shady (C)
Sunny (O)
Deadwood origin
Beech (B)
Fir (F)
Beech + Fir
Canopy (O/C)
Origin of deposited deadwood
Origin of sampled branch (B/F)
Sample type
canopy - origin of FWD pile - DW samples
C - B pile - B FWD
C - B pile - B FWD
C - F pile - F FWD
C - F pile - F FWD
C - mix pile - B FWD
C - mix pile - F FWD
C - mix pile - B FWD
C - mix pile - F FWD
O - B pile - B FWD
O - B pile - B FWD
O - F pile - F FWD
O - F pile - F FWD
O - mix pile - B FWD
O - mix pile - F FWD
O - mix pile - B FWD
O - mix pile - F FWD
6 shady sites
6 sunny sites
4 x beech deadwood
4 x fir deadwood
4 x mixed deadwood
8 x beech sampled
8 x fir sampled
16 x 4 blocks
64 samples
