# Supplementary figures and images for: Fragile foundations: succession patterns of bacterial communities in fine woody debris and soil under long-term microclimate influence
Source: Environ Microbiome. 2025 Aug 6;20:101. doi: 10.1186/s40793-025-00756-9 (PMC12330196; doi:10.1186/s40793-025-00756-9)

# Soil bacteria community structure after 10 years of development under different canopy

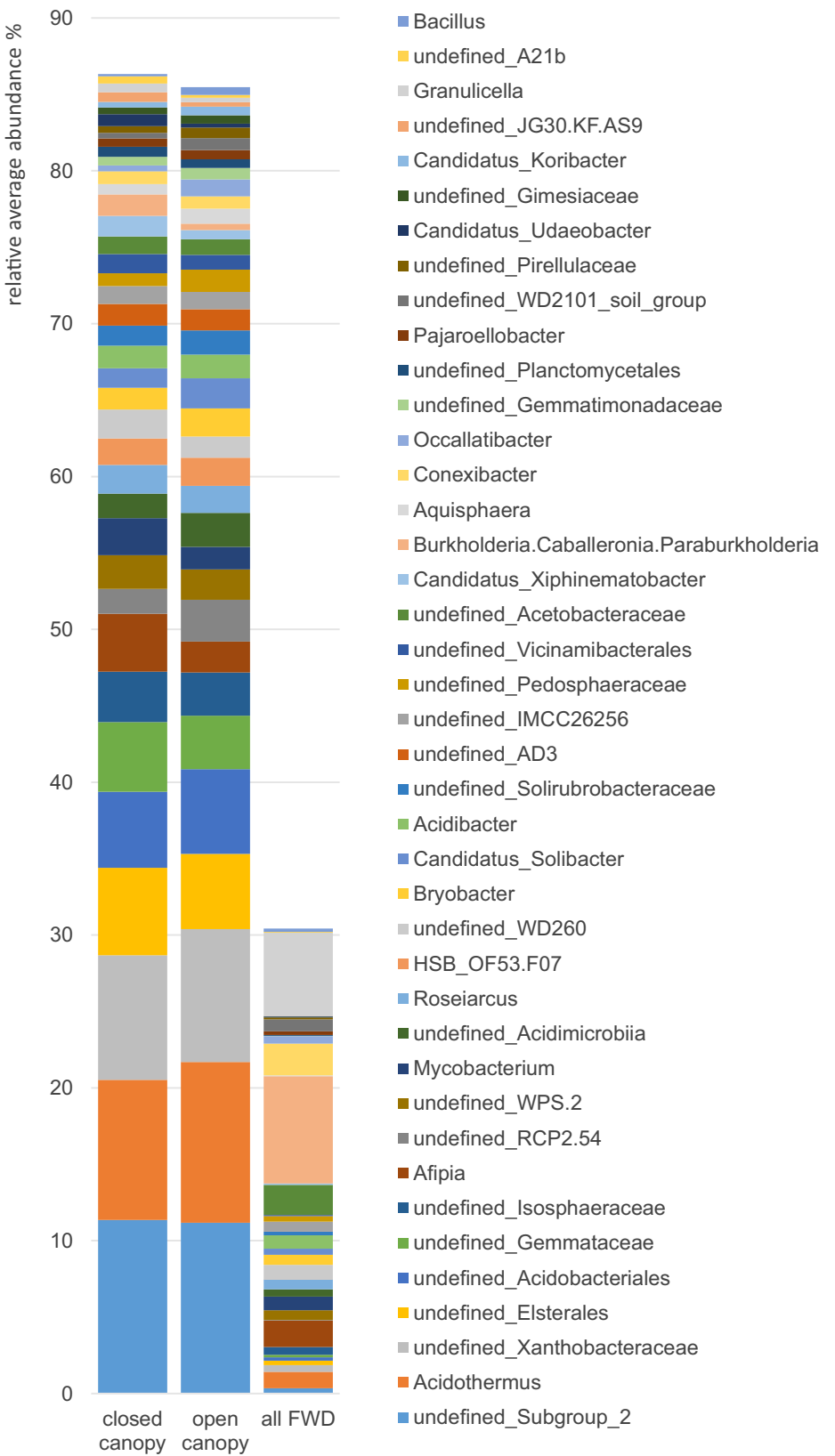

Supplement: Supplementary file 2 — Additional file 2. [file 40793_2025_756_MOESM2_ESM.pdf]
