# Supplementary material for: Fragile foundations: succession patterns of bacterial communities in fine woody debris and soil under long-term microclimate influence
Source: Environ Microbiome. 2025 Aug 6;20:101. doi: 10.1186/s40793-025-00756-9 (PMC12330196; doi:10.1186/s40793-025-00756-9)
Supplement: Supplementary file 3 — Additional file 3. [file 40793_2025_756_MOESM3_ESM.pdf]

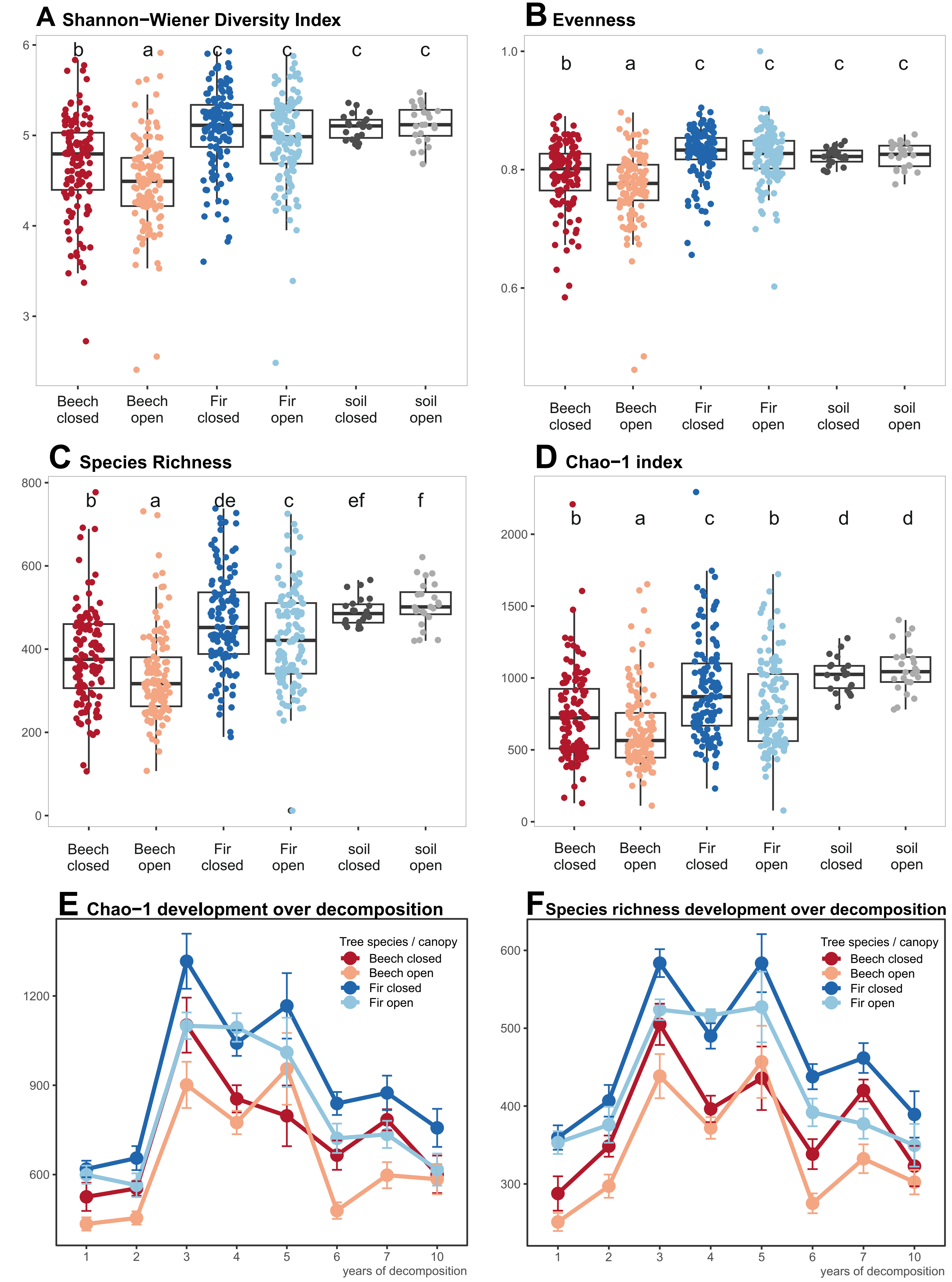

A Shannon−Wiener Diversity Index
B Evenness
C Species Richness
D Chao−1 index
E Chao−1 development over decomposition
F Species richness development over decomposition
Beech closed
Beech open
Fir closed
Fir open
soil closed
soil open
Tree species / canopy
years of decomposition
