# Supplementary material for: Fragile foundations: succession patterns of bacterial communities in fine woody debris and soil under long-term microclimate influence
Source: Environ Microbiome. 2025 Aug 6;20:101. doi: 10.1186/s40793-025-00756-9 (PMC12330196; doi:10.1186/s40793-025-00756-9)
Supplement: Supplementary file 4 — Additional file 4. [file 40793_2025_756_MOESM4_ESM.pdf]

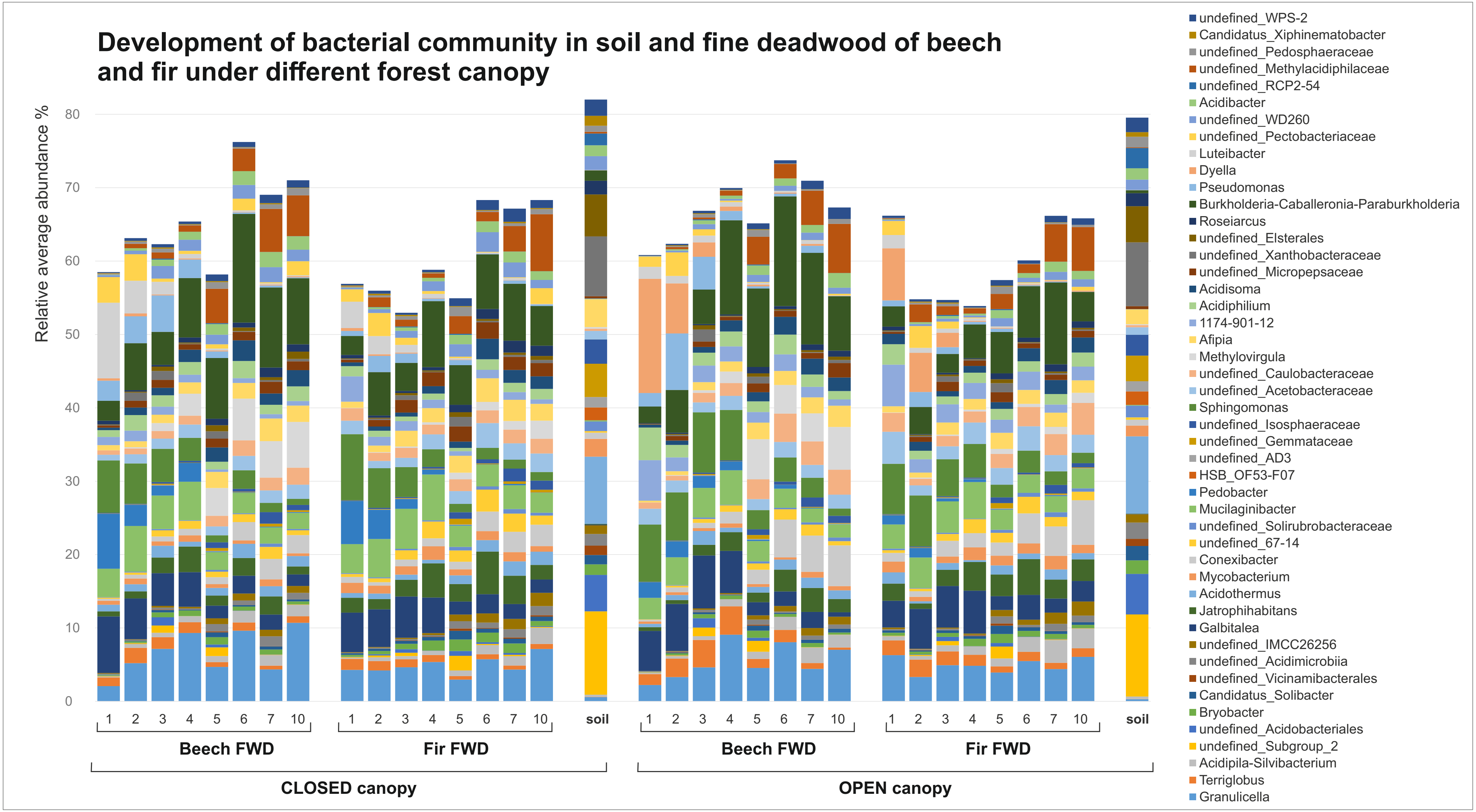

Development of bacterial community in soil and fine deadwood of beech and fir under different forest canopy
Relative average abundance %
0
10
20
30
40
50
60
70
80
1 2 3 4 5 6 7 10
1 2 3 4 5 6 7 10
soil
1 2 3 4 5 6 7 10
1 2 3 4 5 6 7 10
soil
Beech FWD
Fir FWD
Beech FWD
Fir FWD
CLOSED canopy
OPEN canopy
undefined_WPS-2
Candidatus_Xiphinematobacter
undefined_Pedosphaeraceae
undefined_Methylacidiphilaceae
undefined_RCP2-54
Acidibacter
undefined_WD260
undefined_Pectobacteriaceae
Luteibacter
Dyella
Pseudomonas
Burkholderia-Caballeronia-Paraburkholderia
Roseiarcus
undefined_Elsterales
undefined_Xanthobacteraceae
undefined_Micropepsaceae
Acidisoma
Acidiphilium
1174-901-12
Afipia
Methylovirgula
undefined_Caulobacteraceae
undefined_Acetobacteraceae
Sphingomonas
undefined_Isosphaeraceae
undefined_Gemmataceae
undefined_AD3
HSB_OF53-F07
Pedobacter
Mucilaginibacter
undefined_Solirubrobacteraceae
undefined_67-14
Conexibacter
Mycobacterium
Acidothermus
Jatrophihabitans
Galbitalea
undefined_IMCC26256
undefined_Acidimicrobiia
undefined_Vicinamibacterales
Candidatus_Solibacter
Bryobacter
undefined_Acidobacteriales
undefined_Subgroup_2
Acidipila-Silvibacterium
Terriglobus
Granulicella
