# Supplementary material for: Fragile foundations: succession patterns of bacterial communities in fine woody debris and soil under long-term microclimate influence
Source: Environ Microbiome. 2025 Aug 6;20:101. doi: 10.1186/s40793-025-00756-9 (PMC12330196; doi:10.1186/s40793-025-00756-9)
Supplement: Supplementary file 5 — Additional file 5. [file 40793_2025_756_MOESM5_ESM.pdf]

Variable		F value	Pr(>F)	significance
**Carbon content**	**C %**			
	canopy	9.493	0.004	**
	area	2.878	0.049	*
	canopy:area	1.306	0.287	
	soil x FWD	3036	0.000	***
**Nitrogen content**	**N %**	F value	Pr(>F)	significance
	canopy	3.054	0.089	.
	area	3.032	0.041	*
	canopy:area	0.731	0.540	
	soil x FWD	155.8	0.000	***
**CN ratio**		F value	Pr(>F)	significance
	canopy	35.364	0.000	***
	area	1.482	0.235	
	canopy:area	1.854	0.154	
	soil x FWD	606.8	0.000	***
**pH**		F value	Pr(>F)	significance
	canopy	1.033	0.316	
	area	0.41	0.746	
	canopy:area	0.936	0.433	
	soil x FWD	8.964	0.003	**
**ergosterol**	**µg g−1**		Pr(>F)	significance
	canopy	0.478	0.494	
	area	9.046	0.000	***
	canopy:area	0.95	0.426	
	soil x FWD	134.9	0.000	***
**soil moisture**	**%**	F value	Pr(>F)	significance
	canopy	12.648	0.001	**
	area	4.108	0.013	*
	canopy:area	0.93	0.435	
	soil x FWD	35.4	0.000	***

Signif. codes: 0 ‘***’ 0.001 ‘**’ 0.01 ‘*’ 0.05 ‘.’ 0.1 ‘ ’ 1
tested on log10(x+1) transformed data
