# Supplementary material for: Fragile foundations: succession patterns of bacterial communities in fine woody debris and soil under long-term microclimate influence
Source: Environ Microbiome. 2025 Aug 6;20:101. doi: 10.1186/s40793-025-00756-9 (PMC12330196; doi:10.1186/s40793-025-00756-9)
Supplement: Supplementary file 6 — Additional file 6. [file 40793_2025_756_MOESM6_ESM.pdf]

Supplementary table2 : Soil bacteria abundance				Average abundance %				canopy specificity		soil/ FWD specificity	
genus	phylum	class	order	all soil	closed canopy	open canopy	all FWD	1 closed / 0 open		1 soil/ 0 FWD	
undefined_Subgroup_2	Acidobacteriota	Acidobacteriae	Subgroup_2	11.3	11.4	11.2	0.3	0.482		0.756	soil prefering
Acidothermus	Actinobacteriota	Actinobacteria	Frankiales	9.8	9.2	10.5	1.1	0.444		0.465	
undefined_Xanthobacteraceae	Proteobacteria	Alphaproteobacteria	Rhizobiales	8.4	8.2	8.7	0.4	0.462		0.657	
undefined_Elsterales	Proteobacteria	Alphaproteobacteria	Elsterales	5.3	5.7	4.9	0.3	0.516		0.626	
undefined_Acidobacteriales	Acidobacteriota	Acidobacteriae	Acidobacteriales	5.3	5.0	5.5	0.2	0.452		0.702	
undefined_Gemmataceae	Planctomycetota	Planctomycetes	Gemmatales	4.0	4.5	3.5	0.2	0.544		0.687	
undefined_Isosphaeraceae	Planctomycetota	Planctomycetes	Isosphaerales	3.1	3.3	2.8	0.5	0.517		0.365	
Afipia	Proteobacteria	Alphaproteobacteria	Rhizobiales	2.9	3.8	2.0	1.7	0.633		0.137	FWD prefering
undefined_RCP2.54	RCP2-54	undefined_RCP2-54	undefined_RCP2-54	2.2	1.6	2.8	0.0	0.350		0.850	soil prefering
undefined_WPS.2	WPS-2	undefined_WPS-2	undefined_WPS-2	2.1	2.2	2.0	0.6	0.506		0.238	FWD prefering
Mycobacterium	Actinobacteriota	Actinobacteria	Corynebacteriales	1.9	2.4	1.5	0.9	0.603		0.169	FWD prefering
undefined_Acidimicrobiia	Actinobacteriota	Acidimicrobiia	undefined_Acidimicrobiia	1.9	1.6	2.2	0.5	0.396		0.281	
Roseiarcus	Proteobacteria	Alphaproteobacteria	Rhizobiales	1.8	1.9	1.8	0.6	0.491		0.217	FWD prefering
HSB_OF53.F07	Chloroflexi	Ktedonobacteria	Ktedonobacterales	1.8	1.7	1.8	0.0	0.465		0.996	soil
undefined_WD260	Proteobacteria	Gammaproteobacteria	WD260	1.6	1.9	1.4	1.0	0.554		0.137	FWD prefering
Bryobacter	Acidobacteriota	Acidobacteriae	Bryobacterales	1.6	1.4	1.8	0.7	0.416		0.190	FWD prefering
Candidatus_Solibacter	Acidobacteriota	Acidobacteriae	Solibacterales	1.6	1.3	2.0	0.4	0.374		0.285	
Acidibacter	Proteobacteria	Gammaproteobacteria	Gammaproteobacteria_Incertae_Sedis	1.5	1.5	1.5	0.9	0.465		0.142	FWD prefering
undefined_Solirubrobacteraceae	Actinobacteriota	Thermoleophilia	Solirubrobacterales	1.5	1.3	1.6	0.3	0.430		0.353	
undefined_AD3	Chloroflexi	AD3	undefined_AD3	1.4	1.4	1.4	0.0	0.483		0.827	soil prefering
undefined_IMCC26256	Actinobacteriota	Acidimicrobiia	IMCC26256	1.1	1.2	1.1	0.6	0.490		0.153	FWD prefering
undefined_Pedosphaeraceae	Verrucomicrobiota	Verrucomicrobiae	Pedosphaerales	1.1	0.8	1.5	0.4	0.345		0.234	FWD prefering
undefined_Vicinamibacterales	Acidobacteriota	Vicinamibacteria	Vicinamibacterales	1.1	1.3	1.0	0.1	0.546		0.621	
undefined_Acetobacteraceae	Proteobacteria	Alphaproteobacteria	Acetobacterales	1.1	1.1	1.0	2.0	0.506		0.049	FWD
Candidatus_Xiphinematobacter	Verrucomicrobiota	Verrucomicrobiae	Chthoniobacterales	1.0	1.3	0.6	0.1	0.667		0.496	
Burkholderia.Caballeronia.Paraburkholderia	Proteobacteria	Gammaproteobacteria	Burkholderiales	0.9	1.4	0.4	7.0	0.758	closed prefering	0.012	FWD
Aquisphaera	Planctomycetota	Planctomycetes	Isosphaerales	0.8	0.7	1.0	0.1	0.386		0.569	
Conexibacter	Actinobacteriota	Thermoleophilia	Solirubrobacterales	0.8	0.8	0.8	2.1	0.488		0.037	FWD
Occallatibacter	Acidobacteriota	Acidobacteriae	Acidobacteriales	0.7	0.4	1.1	0.5	0.246	open prefering	0.134	FWD prefering
undefined_Gemmatimonadaceae	Gemmatimonadota	Gemmatimonadetes	Gemmatimonadales	0.7	0.6	0.8	0.0	0.401		0.665	
undefined_Planctomycetales	Planctomycetota	Planctomycetes	Planctomycetales	0.6	0.7	0.6	0.0	0.520		0.583	
Pajaroellobacter	Myxococcota	Polyangia	Polyangiales	0.6	0.6	0.6	0.2	0.453		0.189	FWD prefering
undefined_WD2101_soil_group	Planctomycetota	Phycisphaerae	Tepidisphaerales	0.6	0.4	0.8	0.8	0.300		0.067	FWD prefering
undefined_Pirellulaceae	Planctomycetota	Planctomycetes	Pirellulales	0.6	0.4	0.7	0.1	0.370		0.280	
Candidatus_Udaeobacter	Verrucomicrobiota	Verrucomicrobiae	Chthoniobacterales	0.5	0.8	0.3	0.0	0.730		0.552	
undefined_Gimesiaceae	Planctomycetota	Planctomycetes	Planctomycetales	0.5	0.4	0.5	0.0	0.429		0.646	
Candidatus_Koribacter	Acidobacteriota	Acidobacteriae	Acidobacteriales	0.5	0.4	0.6	0.0	0.368		0.719	
undefined_JG30.KF.AS9	Chloroflexi	Ktedonobacteria	Ktedonobacterales	0.5	0.6	0.3	0.0	0.667		0.968	soil
Granulicella	Acidobacteriota	Acidobacteriae	Acidobacteriales	0.4	0.6	0.3	5.5	0.644		0.007	FWD
undefined_A21b	Proteobacteria	Gammaproteobacteria	Burkholderiales	0.3	0.5	0.2	0.1	0.703		0.328	
Bacillus	Firmicutes	Bacilli	Bacillales	0.3	0.1	0.5	0.2	0.200	open prefering	0.127	FWD prefering

Specificity cut off: 95% of total number of sequences present in one of the groups, value reflect the ratio of sequence abundance sum under closed canopy.
If over 75% of sequences were detected in any of groups, signed as "prefered".
